# PD60 The Importance Of Systematic Reviews Addressing Questions Of Prevalence In Health Technology Assessment

**DOI:** 10.1017/S0266462324003180

**Published:** 2025-01-07

**Authors:** Celina Borges Migliavaca, Timothy Hugh Barker, Cinara Stein, Verônica Colpani, Zachary Munn, Maicon Falavigna

## Abstract

**Introduction:**

The use of systematic reviews (SRs) of interventions is commonplace in health technology assessment (HTA). However, SRs synthesizing other data types, such as prevalence, are rarely used. These SRs may complement the HTA process by gathering complementary evidence essential for developing trustworthy recommendations. We aimed to discuss the importance and application of SRs of prevalence in the context of HTA.

**Methods:**

A methodological working group, the Prevalence Estimates Reviews – Systematic Review Methodology Group (PERSyst), was created to provide guidance on how to improve the development of SRs and meta-analyses of prevalence. As part of the group’s work, a guide for HTA developers regarding the value of SRs of prevalence was developed.

**Results:**

There are many benefits to including SRs of prevalence in the process of HTA. These include providing data for estimating burden of disease; helping to set priorities regarding technology assessment; informing the absolute impact on health outcomes from association measures (e.g., relative risk) reported in clinical studies; and providing data for estimating resource requirements for and feasibility of implementing health technologies under consideration. Within the GRADE framework, prevalence estimates are necessary to assess the quality of diagnostic test accuracy evidence and to support decision-making using the Evidence to Decision framework.

**Conclusions:**

Although not commonly used, SRs of prevalence are an important tool in the process of HTA. There is a need for standardization of methodologies and guidance on how to use these reviews in the HTA process.

